# The Government of Kenya's Cash Transfer Program Reduces the Risk of Sexual Debut among Young People Age 15-25

**DOI:** 10.1371/journal.pone.0085473

**Published:** 2014-01-15

**Authors:** Sudhanshu Handa, Carolyn Tucker Halpern, Audrey Pettifor, Harsha Thirumurthy

**Affiliations:** 1 Carolina Population Center and Department of Public Policy, University of North Carolina, Chapel Hill, North Carolina, United States of America; 2 Carolina Population Center and Department of Maternal and Child Health, Gillings School of Global Public Health, University of North Carolina, Chapel Hill, North Carolina, United States of America; 3 Carolina Population Center and Department of Epidemiology, Gillings School of Global Public Health, University of North Carolina, Chapel Hill, North Carolina, United States of America; 4 Carolina Population Center and Department of Health Policy and Management, Gillings School of Global Public Health, University of North Carolina, Chapel Hill, North Carolina, United States of America; University of Southampton, United Kingdom

## Abstract

The aim of this study is to assess whether the Government of Kenya's Cash Transfer for Orphans and Vulnerable Children (Kenya CT-OVC) can reduce the risk of HIV among young people by postponing sexual debut. The program provides an unconditional transfer of US$20 per month directly to the main caregiver in the household. An evaluation of the program was implemented in 2007–2009 in seven districts. Fourteen Locations were randomly assigned to receive the program and fourteen were assigned to a control arm. A sample of households was enrolled in the evaluation in 2007. We revisited these households in 2011 and collected information on sexual activity among individuals between 15–25 years of age. We used logistic regression, adjusted for the respondent's age, sex and relationship to caregiver, the age, sex and schooling of the caregiver and whether or not the household lived in Nairobi at baseline, to compare rates of sexual debut among young people living in program households with those living in control households who had not yet entered the program. Our results, adjusted for these covariates, show that the program reduced the odds of sexual debut by 31 percent. There were no statistically significant effects on secondary outcomes of behavioral risk such as condom use, number of partners and transactional sex. Since the CT-OVC provides cash to the caregiver and not to the child, and there are no explicit conditions associated with receipt, these impacts are indirect, and may have been achieved by keeping young people in school. Our results suggest that large-scale national social cash transfer programs with poverty alleviation objectives may have potential positive spillover benefits in terms of reducing HIV risk among young people in Eastern and Southern Africa.

## Introduction

There is growing interest in the possibility of addressing HIV prevention in Africa by providing cash transfers to at-risk population groups [1]. A recent review identified no less than 16 completed or on-going studies that seek to measure the impact of cash transfers on HIV behavioral risk or HIV status [2]. Most of these studies examine cash transfer interventions that target adolescents and can be grouped into one of two types: 1) those whose primary goal is child protection and/or poverty alleviation; and 2) those with an explicit health objective and that condition payments on achievement of specific behaviors (such as testing HIV-negative). Of the studies reviewed, 9/10 have shown positive results on sexual behavior, and one has shown a reduction in HIV prevalence, with lower prevalence among young women in the intervention relative to a control group [3].

Collectively the evidence supports the concept that cash transfers may reduce the risk of HIV by addressing structural risk factors, specifically poverty, vulnerability and low human capital. However, the studies conducted to date are pilots or localized experiments with different program parameters and target populations. In contrast, almost all countries (n = 14) in Eastern & Southern Africa (ESA) have large-scale government cash transfer programs that target poor and vulnerable households, and are driven by poverty alleviation objectives rather than HIV prevention. These programs provide unconditional cash transfers meaning that they do not include conditions related to behavioral change in order for eligible households to receive payments. This raises the question about whether the existing evidence on the HIV prevention effects of cash transfers can be generalized to large-scale, government-run cash transfer programs currently operating in the region.

Here we examine the impact of the Government of Kenya's Cash Transfer for Orphans and Vulnerable Children program (CT-OVC), the largest social protection program in Kenya, on sexual debut among young people ages 15–25 years. This program is similar in terms of objective, targeting, and program design parameters to large-scale programs operated by governments across Eastern and Southern Africa, and so results from this study are more likely to be relevant for other cash transfer programs in the region. In particular, the results from this study can provide evidence on whether poverty targeted cash transfer programs that give cash to caregivers rather than children and which do not explicitly have HIV prevention objectives can nonetheless reduce the risk of HIV by delaying sexual debut among young people in beneficiary households.

### Cash Transfer and HIV Risk

Given the generalized prevalence of HIV in ESA and the idea that poverty may be an important structural determinant of HIV risk, the public health community has become increasingly interested in the potential for monetary incentives to reduce HIV related risk behavior among young people. A conceptual framework for this link is offered in Medlin & de Walque [4] and several recent studies have tested the hypothesis that *conditional* cash payments can reduce HIV risk. Baird et al. [3] conducted a randomized control trial (RCT) of cash transfers to young women in Malawi that were conditional on school enrollment and reported a decrease in the prevalence of HIV and herpes simplex 2. De Walque et al. [5] conducted an RCT among individuals age 18–30 in rural Tanzania and found limited evidence that a cash transfer conditional on testing negative for four sexually transmitted infections reduced the prevalence of testing positive for their combined prevalence, with slightly higher payments associated with larger effects. A similar experiment in Lesotho which linked lottery tickets to maintaining negative for two STIs reported lower incidence of HIV among lottery winners [6]. In contrast Kohler & Thornton [7] report no impact on maintaining HIV-negative status from an experimental conditional cash transfer (CCT) conducted in Malawi, though in their case the cash transfer was provided at the end of the trial (one year later) rather than during the trial itself. Finally, a 3-year RCT of a school-based CCT in rural Zimbabwe found that comprehensive school support was effective in reducing early marriage among orphan girls (one or both parents deceased) who were in grade 6 at study entry [8].

There are several pathways through which a poverty-targeted cash transfer program could delay sexual debut. Foremost among these is that cash transfers can increase school enrollment and attainment, which can reduce HIV related risky behavior [9] [10]. School provides an environment where sex is less likely to occur and increases the likelihood of partnering with potential sex partners who are closer in age, and thus less likely to be HIV infected. School attendance may also increase exposure to HIV education and may enhance an adolescent's ability to effectively act on HIV prevention information received due to greater cognitive function. The Kenya CT-OVC program studied here has had a significant impact on increasing school enrollment; secondary school age children in program households were 8 percentage points more likely to be enrolled in school relative to the control group [11]. Educational attainment has been found to be associated with a lower risk of HIV infection in a number of high HIV prevalence settings [12] [10], while in longitudinal cohort studies conducted in Zambia and Uganda, HIV prevalence has declined most rapidly among young people with the most education [12] [13]. Another pathway through which cash transfer programs may delay sexual debut is through increased economic well-being. Poorer individuals may be at increased risk through mechanisms similar to those hypothesized that put individuals with less education at increased risk. In addition, young people of lower economic status may be more likely to engage in transactional sex (for goods such as food, housing, social status or money), increasing their HIV risk.

Interventions to delay sexual debut have been an important part of the HIV prevention strategy in sub-Saharan Africa (SSA) due to the association between age at first sex and HIV infection [14] [15]. A review of 26 studies from SSA which included biological tests for HIV has recently been conducted by Stockl et al. [16]. They report that “among high quality studies, there is consistent evidence of an association between early sex and HIV risk, which remained after several potential confounders were adjusted for (page 35).” The authors also looked for evidence supporting several possible pathways underlying the link − they report little or no evidence for longer duration of sexual activity or the older partner hypothesis, but some evidence to support greater sexual risk taking as a pathway.

## Methods

### The CT-OVC program

The CT-OVC is implemented by the Children's Department of the Ministry of Gender, Children and Social Development of the Government of Kenya and targets households that are poor and have at least one orphan (single or double orphan) or vulnerable child below 18 years of age. The program provides a flat transfer of approximately USD20 (at an exchange rate of US$1:KES75 in 2007) per month (paid bi-monthly) given directly to the caregiver to allow these households to provide for the care and support of OVC. The program was pilot-tested in 2004-2006 and began phased expansion in 2007. While there are currently no punitive conditions associated with the program, recipients are informed that the money is to be used for the care of OVC living in the household. As of February 2012 the program reached 134,000 households and approximately 280,000 OVC across Kenya. The amount provided per month and household selection procedures are described in Table 1. Participants in the evaluation are a sample of households that were part of the expansion phase of the program in 2007.

**Table 1:** Program Details of the Kenya Cash Transfer for Orphans and Vulnerable Children.

**Beneficiary Population**
Poor households across Kenya containing at least one OVC age 0–17. An orphan is defined as any child with at least one biological parent deceased. A vulnerable child is one who is either chronically ill or whose main caregiver is chronically ill. Beneficiary selection is done in two stages. To satisfy the poverty criteria households must display 8 out of 13 characteristics related to welfare such as main material of walls and floors, access to potable water, type of lighting fuel, and ownership of small assets.
**Targeting**
In Stage 1, OVC Committees in each Location (4^th^ administrative unit below province, district and division, consisting of a group of communities) identify potentially eligible households based on poverty and demographic criteria. In Stage 2, listed households are enumerated by Ministry staff to confirm poverty status. Households are then prioritized by age of head, with child-headed households prioritized first followed by older heads.
**Intervention**
Kenya Shilling (KES)1500 (US$20) per month transfer irrespective of household size, paid bimonthly directly to the caregiver. Payment is not conditional on any child or adult behaviors, although caregivers are instructed that receipt of the money is for the care and protection of OVC. The transfer level was increased to KES 2000 in July 2012, after this study was completed.
**Current Scale and Budget**
134,000 households enrolled as of February 2012. FY 2011/12 program budget is KES3.5billion, of which 31 percent is from general tax revenues, 37 percent from development loans and 31 percent from foreign aid donations. The program budget represents less than half a percent of the overall national budget.

### The CT-OVC impact evaluation

During the initial rollout of the CT-OVC in 2007, UNICEF and GOK designed a quantitative study to assess the impact of the program on the primary indicators of per capita consumption, school enrollment, and access to health care. Seven districts were selected to be part of the initial expansion based on overall poverty, level of development, and OVC prevalence. Within each of the seven districts, four Locations were identified to potentially be included in the program though not all could be enrolled at that time. Two Locations in each district were thus selected randomly by lottery to receive the cash transfers immediately and the remaining two Locations served as the control group. Randomization was conducted at the level of Location rather than the community because program implementation functions are delegated to the Location and it is thus the lowest administrative level for the program. A Location consists of up to a dozen communities and can be geographically large and quite diverse. Targeting of households was conducted according to established program guidelines in all intervention Locations, while in control Locations stage 1 and 2 targeting was implemented (see Table 1). Households were masked at baseline to reduce the possibility of anticipation effects (where participants change their behavior in anticipation of receiving the transfer). Due to the nature of the intervention, participants in the study were not masked after baseline.

From the complete eligibility list in the control Locations and the prioritized eligibility list in the intervention locations, households were randomly selected to enter into the evaluation study with a sampling fraction of 1 (control):2 (intervention). Households were assigned a computer generated number (within each Location), then sorted in ascending order by this number and selected for the study in this order until the desired sample size was reached in each Location. Minimum sample sizes were determined on the basis of power calculations to be able to observe a change of 5% in school enrollment, 20% in curative health care, and 10% in per capita consumption (The primary indicators for the program) accounting for possible intra-cluster correlation at the community level. It is important to note that since the objective of the program was not HIV prevention, the sample size for the evaluation was not based on the indicators presented in this study. In total 1540 and 754 households were selected from the intervention and control Locations respectively. A household survey was administered at baseline in 2007 (wave 1) and again in 2009 (wave 2) and collected and analyzed by Oxford Policy Management (OPM), under contract to UNICEF [17].

### The follow-up evaluation

**Figure 1:**
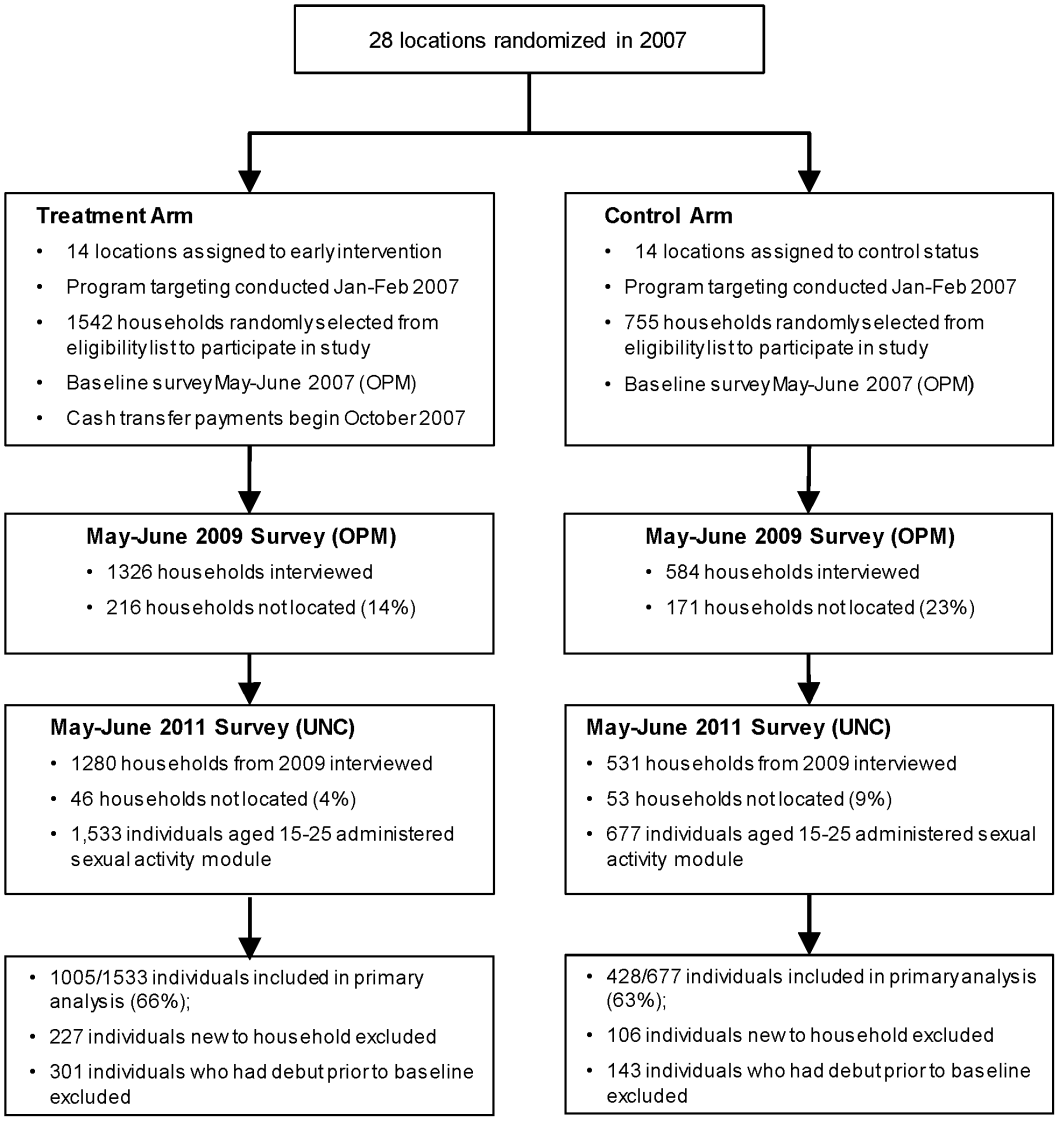
Overview of Study Design.

In 2011 the authors of this study collected a third wave of data from the same households in the original evaluation sample who were interviewed in both 2007 and 2009. In addition to the basic household questionnaire, we included a module on sexual behavior to be administered to up to three household members aged 15–25. If more than three individuals were eligible, research assistants were instructed to interview the three youngest individuals in order to prioritize those least likely to have had their sexual debut. The survey instrument was based on the Kenya Demographic and Health Survey (KDHS) [18] and contained questions on sexual experience, condom use, transactional sex and partner characteristics. Since this module was only administered during the third wave in 2011, we do not have pre-program information on these variables. Figure 1 depicts the study design and participant flows. As of mid-2013 the program had not scaled up completely in the evaluation districts hence the original control group has yet to enter the program.

### Ethics statement

Our survey protocols were similar to that of the KDHS. All respondents were invited to participate in the survey after receiving an explanation of the study objectives, procedures, risks and benefits and provided written informed consent. For children ages 15–17, we sought written informed consent from the parent or main caregiver and assent from the child. All interviews were conducted by same-sex interviewers in a private place; the interview was terminated if privacy could not be assured. Interviews were conducted in either Luo, Swahili or Somali depending on the region. Study protocols, including consent procedures, were approved by the Kenya Medical Research Institute Ethics Review Committee (Protocol #265) and the Institutional Review Board of the University of North Carolina.

### Attrition

The current study is based on data that were collected by UNC in 2011 as a separate follow-up to the original data collection in 2007–2009. That initial data collection period coincided with a time of political turmoil in Kenya that resulted from the disputed national elections in December 2007 and led to the internal displacement of over 400,000 individuals. Consequently, attrition between baseline and the first follow-up in 2009 was 17 percent and concentrated in Kisumu and Nairobi, the two Locations in the study that experienced the most election-related unrest. Attrition between the 2009 and 2011 rounds which UNC was engaged in was only five per cent. Table 2 shows means of selected demographic and poverty measures for household in each arm across the three waves. Means for these indicators are stable across the three waves despite the relatively high attrition rate between 2007 and 2009, indicating that the representativeness of the sample remained intact.

**Table 2:** Mean household characteristics in Kenya CT-OVC evaluation by wave and intervention status.

Sample:	2007		2009		2011	
	T	C	T	C	T	C
Demographics						
Household size	5.48	5.79	5.54	5.81	5.53	5.82
Female head	0.65	**0.57** 1	0.65	**0.59** 1	0.65	**0.59** 1
Age of head in years	62.34	**56.06** 1	62.21	**56.20** 1	62.55	**56.55** 1
Head not completed primary	0.53	**0.38** 1	0.53	**0.38** 1	0.53	**0.38** 1
Poverty						
Per adult equiv. monthly consumption (KS)	1533	1501	1542	**1460**	1550	**1442**
Walls of mud/dung/grass/sticks	0.75	0.84	0.75	0.86	0.74	0.87
Roof of mud/dung/grass/sticks	0.23	0.22	0.23	0.23	0.22	0.22
Floor of mud/dung	0.66	0.74	0.65	0.77	0.66	0.79
No toilet	0.55	0.56	0.55	0.56	0.54	0.56
Unprotected water source	0.62	0.68	0.61	0.70	0.61	0.70
N	1540	754	1325	583	1266	545

^1^ Statistically significant (at 5%) differences of t-test between Treatment (T) and Control (C) within each wave shown in bold.

We provide a detailed analysis of the determinants of attrition as supporting information in Table S1 and describe the main results of that analysis here. We estimated the probability of attriting between 2007 and 2009 using baseline values for the variables reported in Table 2 plus district dummy variables, the number of residents in each of five age categories and the log of household size. The only statistically significant variables out of the 26 total variables in this regression were the indicators for Kisumu and Nairobi, the number of residents age 12–17, log of household size and unprotected water source. The probability of attriting was 19 and nine percentage points higher for households in Nairobi and Kisumu respectively (relative to the reference), while households in the intervention group were eight points less likely to be lost at follow-up relative to the control group. We looked for *differential* determinants of attrition between the two groups by re-estimating this model interacting each regressor with the indicator for intervention status. In only two cases (out of a possible 26) was there a statistically significant interaction effect (the indicator for residence in Kwale District, and the number of residents age 6–11). Based on the stability of characteristics in each arm across the waves, the fact that the two most important determinants of attrition stem from residence in Kisumu and Nairobi, which were disproportionately affected by the election violence relative to other study sites, and the minimal differences in the determinants of attrition across arms, non-random attrition is likely to be small.

### Response rates among adolescent sample

A total of 2210 out of a possible 2797 individuals age 15–25 were interviewed. Of the 588 individuals not interviewed, 184 were excluded because they were the oldest in a household with more than three members in the target group and so were purposefully excluded, leaving an actual response rate of 85 percent. The most common reason for non-response was because respondents were in boarding school or away from home during the study period due to work or other reasons. The response rate is higher in the control arm (87 versus 83 percent), which is consistent with the fact that the CT-OVC has had a positive impact on secondary school enrollment and individuals in treatment households were thus more likely to be away in boarding school.

We performed several checks to understand the potential implications of the non-response rate for our results. First we compared the age and sex of respondents versus those that should have been interviewed but were not, and found no statistical differences between the two groups. However non-respondents were four percentage points more likely to be enrolled in school relative to respondents (66 versus 62 percent). We also compared the age, sex and schooling of the head of household of respondents and non-respondents, as well as per capita household expenditure (a measure of economic well-being) and did not find any statistically significant differences. If part of the lower response rate among individuals in treatment households is explained by presence at boarding schools, and if attendance at boarding schools is protective of sexual debut, then we may have a small downward bias in the program effect due to selective non-response.

### Outcomes

The primary outcome variable of this study was a self-report of whether the respondent had ever had vaginal intercourse. We also defined four secondary outcomes for those who had their sexual debut after baseline. We report on whether or not a condom was used at last sex, whether or not they had ever received or given gifts in exchange for sex, whether or not the respondent had two or more different partners in the last 12 months and whether or not the respondent had any unprotected sex acts in the last three months.

### Statistical analysis

Our objective was to determine the effect of living in a beneficiary household on self-reported sexual debut four years after program initiation. Since we do not have a pre-intervention measure of the primary outcome variable we excluded all respondents who had their sexual debut prior to baseline since the program could not affect this outcome for such individuals. This represented 301 (or 23 percent) and 143 (25 percent) individuals in the treatment and control arms respectively. In this group we compare rates of sexual debut between treatment and control arms four years after intervention households began receiving the cash transfer. We further restricted our sample to individuals who had been living in the household for at least two years to ensure they had a minimum level of exposure to the intervention. We compute odds ratios using logistic models that regress the outcome variable, sexual debut, against a treatment status indicator, age (in years) and sex of the respondent, and age, sex and schooling of the head of household, as well as an indicator for whether or not the respondent lives in Nairobi, all measured at baseline values except for respondent age. The choice of control variables is based on whether or not they were balanced at baseline. We follow the analysis method of the original evaluation [11] [17], and adjust the standard errors of individual outcomes for clustering at the household level since there are multiple respondents per household and this is considered to be the strongest source of homogeneity for individual level outcomes. In our sample there is an average of 1.4 respondents per household and the intra-cluster correlation for the primary outcome (vaginal intercourse) is 0.08. Stata Version 12 was used for all the statistical analysis.

## Results

Table 2 shows mean values of key household characteristics in each of the three waves of data collection. Households are extremely poor, with a mean per adult equivalent monthly consumption expenditures of approximately US$20 per month or 60 cents per day. Most households did not have a protected water source, and live in homes with walls made of mud, dung or grass.

Table 3 presents means of our primary and secondary indicators of HIV behavioral risk among the full analysis sample. Of the 1,443 individuals in the sample, 70 percent were in the treatment arm and 61 percent were male. As mentioned earlier the male-female ratio reported in Table 3 mirrors that of the full sample of residents age 15–25 and is the same as the ratio among those aged 11–21 at baseline (four years earlier). Thus it is not a result of either differential attrition or non-response but reflects the underlying M/F ratio among these types of households. The rate of sexual debut in the treatment group is 38 percent versus 44 percent in the control group (p-value = 0.001), and condom use at last sex is also higher at 43 percent versus 39 percent in the control group. Indeed all behaviors appear to be more protective among the treatment group, though none are statistically different from the control group except for sexual debut.

**Table 3:** Summary statistics of outcomes for young people 15–25 year olds in 2011 in Kenya.

	Treatment		Control		
	Mean or N	SD or %	Mean or N	SD or %	P-value for difference
Number of Individuals	1005		428		
Age in years (N = 1433)	17.69	2.29	17.87	2.30	0.164
Female (N = 1433)	553	38.41	167	39.02	0.837
Vaginal Intercourse (N = 1433)	361	35.92	190	44.39	0.001
Condom at Last Sex (N = 551)	155	42.94	75	39.47	0.442
2+ Partners L12 Months (N = 551)	18	4.99	15	7.89	0.213
Any Unprotected Act L3 Months (N = 551)	30	8.31	18	9.47	0.641
Ever Received/Given Gifts (N = 500)^1^	44	13.54	26	14.86	0.703

Numbers are means with SD for continuous variables or sample size with % for binary variables. Last column shows p-value for difference in mean or proportion. Sample restricted to those who had not yet had sex at baseline.

^1^ 51 observations with missing values.

Table 4 presents the main results of this study—the effect of the Kenya CT-OVC on sexual debut. The first set of columns shows the impact of the program on the full sample of males and females 15–25 years. Those in treatment households had significantly lower odds of having initiated vaginal intercourse since baseline than young people in control households (adjusted odds ratio, AOR, 0.69; CI, 0.53 – 0.89). Note that adjusting for covariates hardly changes the effect size. The prevalence ratio associated with these estimates is 0.82; alternatively the effect size in terms of percentage points is 8.8 which is 23 percent of the baseline mean. The estimates further imply about a half year delay in sexual debut.

**Table 4:** Effect of Kenya CT-OVC on sexual debut among males and females 15–25 years in 2011.

	All		Females		Males	
	Unadjusted	Adjusted	Unadjusted	Adjusted	Unadjusted	Adjusted
Household in CT-OVC	0.702	0.689	0.581	0.576	0.788	0.742
95% CI	(0.555 – 0.889)	(0.531 – 0.893)	(0.393 – 0.858)	(0.383 – 0.868)	(0.585 – 1.061)	(0.531 – 1.037)
P-value	0.003	0.005	0.006	0.008	0.117	0.081
Age of head (years)		1.004		1.003		1.004
95% CI		(0.997 – 1.010)		(0.993 – 1.014)		(0.995 – 1.012)
P-value		0.306		0.535		0.383
Head is female		1.414		1.984		1.146
95% CI		(1.097 – 1.822)		(1.299 – 3.029)		(0.832 – 1.579)
P-value		0.007		0.002		0.404
Head not completed primary school		1.045		1.042		1.045
95% CI		(1.015 – 1.076)		(0.994 – 1.093)		(1.006 – 1.086)
P-value		0.003		0.085		0.023
Respondent is child of head		0.954		0.755		1.065
95% CI		(0.683 – 1.331)		(0.464 – 1.227)		(0.682 – 1.662)
P-value		0.781		0.257		0.782
Respondent is grandchild of head		1.350		0.667		1.959
95% CI		(0.931 – 1.957)		(0.379 – 1.175)		(1.191 – 3.224)
P-value		0.113		0.161		0.008
Nairobi residence		0.792		0.603		1.001
95% CI		(0.546 – 1.151)		(0.345 – 1.055)		(0.605 – 1.657)
P-value		0.222		0.076		0.997
Respondent female		0.877				
95% CI		(0.693 – 1.109)				
P-value		0.273				
Respondent age (years)		1.265		1.260		1.277
95% CI		(1.196 – 1.338)		(1.153 – 1.377)		(1.189 – 1.371)
P-value		<0.001		<0.001		<0.001
Observations	1,433		553		880	

Each column is a logistic regression model estimated on individual data and the dependent variable equals 1 if respondent has had sex and 0 otherwise. Odds ratios (OR) for each independent variable reported in corresponding row. Sample restricted to those who had not yet had sex at baseline.

Subsequent columns show results for females and males, respectively, and indicate that effect sizes are somewhat larger for females with an AOR of 0.58 compared to 0.74 for males and the 95% CI for males now includes 1. Pooling the data and adding an interaction between sex of respondent and treatment status yields an odds-ratio with a p-value of 0.17, hence the effects of the program do not differ statistically by sex.

Table 5 reports the program effect for other HIV behavioral risks. These outcomes are naturally estimated on the 551 individuals who had their sexual debut after the baseline survey. For the full sample presented in the top panel of Table 5, the program appears to have no significant association with any of these secondary outcomes. The two subsequent panels report results by sex. In all cases except for having 2+ partners in the last 12 months for females, the 95% CI for the point estimates include 1. The one statistically significant estimate is driven by a difference of only one less occurrence among females in the treatment group and so cannot be considered a robust result. Given the small sample sizes and resulting large CIs it is illustrative to observe the pattern in the point estimates of the secondary outcomes. Among females, all point estimates suggest that the CT-OVC is protective of these secondary outcomes, while among males three of four are protective.

**Table 5:** Effect of Kenya CT-OVC on other sexual outcomes among individuals 15–25 years in 2011 who reported sexual debut (N = 551).

	Condom at Last Sex	Ever Received or Given Gifts^1^	2+ Partners Last 12 Months	Had Unprotected Sex Last 3 months
	Full Sample			
Adjusted OR	1.199	0.843	0.584	0.901
95% CI	(0.826 – 1.741)	(0.461 – 1.539)	(0.262 – 1.305)	(0.473 – 1.717)
P-value	0.340	0.577	0.190	0.751
Proportion of dep. variable (%)	41.74	14.00	5.99	8.71
Observations	551	500	551	551
	Females			
Adjusted OR	1.330	0.979	0.204	0.650
95% CI	(0.704 – 2.510)	(0.439 – 2.186)	(0.0441 – 0.942)	(0.219 – 1.931)
P-value	0.380	0.959	0.042	0.438
Proportion of dep. variable (%)	36.68	21.2	2.51	8.54
Observations	199	184	199	199
	Males			
Adjusted OR	1.075	0.711	0.686	1.201
95% CI	(0.669 – 1.726)	(0.295 – 1.713)	(0.281 – 1.673)	(0.512 – 2.822)
P-value	0.765	0.447	0.407	0.673
Proportion of dep. variable (%)	44.6	9.81	7.95	8.81
Observations	352	316	352	352

Adjusted odds ratio (OR) calculated with a logistic model on individual data with independent variables that include treatment status, age and sex of respondent (full sample only), age, sex and education of household head and indicator for Nairobi residence. Sample restricted to individuals who had not yet had sex at baseline.

^1^ 51 missing observations for this outcome, 15 females and 36 males.

## Discussion

Our results show that a major government social welfare program that is intended to alleviate poverty, the Kenya CT-OVC, reduces the relative odds of sexual debut among young people ages 15–25 by 31%, with larger impacts among females (42%) relative to males (26%). In relation to the mean, this implies a 23 percent reduction in the likelihood of sexual debut among the full sample, and 35 and 18 percent for females and males respectively. These results are particularly promising for HIV prevention because of their potential generalizability to other large-scale national programs across Africa, and because they demonstrate that a poverty-focused social protection program can have positive spillover effects on one important HIV related outcome. In addition, the effects reported here are for all children in the household and not just OVC.

Beyond sexual debut we did not find any statistically significant program effects on other HIV risk related behaviors such as condom use, number of partners and transactional sex. However the sample sizes for these secondary outcomes are small, and all point estimates indicate that the program is protective for these outcomes, suggesting that a study explicitly powered for these secondary outcomes may well find statistically significant impacts. On the other hand, it is also plausible that an unconditional cash transfer without any explicit messaging around sexual behavior may not be sufficient to affect behaviors beyond age at first sex.

Several studies have established that under certain circumstances, a cash transfer can influence HIV related behavioral risk [8] [19] [20]. Unlike the cash transfer programs assessed in these studies which were conditional cash transfer programs, the Kenya CT-OVC is a government social welfare program with eligibility rules and program parameters that are similar to other large scale government social welfare programs operating in Eastern & Southern Africa including Zambia, Zimbabwe, Malawi and Uganda. Kenya's CT-OVC and these other programs are all unconditional cash transfer programs (or ‘social’ cash transfers) that have the advantage of being less expensive to implement than the conditional cash transfer programs that require monitoring school attendance or other behaviors. Moreover, in contrast to the CCT that was found to have an HIV prevention benefit [3], these programs provide money to heads of household or caretakers of children and not children themselves – the latter being a feature that may not be easily scalable. The results in this study are encouraging as they are generated by a program that had no conditionality, thus was easier to implement, and yet influenced behavior. Indeed the effect size reported here for sexual debut (23 percent) is much larger than the 4 percent reported by Baird [19].

Understanding the mechanisms through which cash transfer programs work to reduce HIV risk is important to ensuring programs can be tailored to achieve maximum impacts. Potential mechanisms for cash transfer program to reduce risk include reducing financial barriers to schooling, thus increasing school attendance and thereby reducing HIV risk [10] [21]. It has also been hypothesized that alleviating poverty may reduce the need for young people to engage in transactional sex which may reduce the risk of HIV. For young women in particular, cash transfer programs may reduce their dependence on male partners, potentially reducing unwanted sexual relationships or unprotected sex acts. It is also possible that lifting homes out of severe poverty improves mental health and increases hope for the future, which may also have protective effects on HIV risk behaviors. It is important that future evaluations of cash transfers programs explore these mechanisms. We are currently conducting analyses to try and understand some of the pathways through which reduced risk behaviors were achieved in the Kenya CT-OVC program, which we will report separately.

This study has several limitations that warrant discussion. First, the study relied on self-reported sexual behavior measures, which may have social desirability bias and raises the possibility that the effects on actual behavior and HIV risk may differ. A number of studies have explored avenues of reducing bias in reporting in developing countries, particularly among adolescents, such as self-administered or computer assisted interviews [22] [23] [24] [25] [26]. Taken together, findings are mixed, and indicate that particularly in resource poor settings where individuals have low exposure to technology, it is not clear that self-administered surveys outperform interviewer administered surveys. However, existing studies from developing countries suggest that there will be more bias among males [27] [25] [28] [29] and older adolescents [28]. However, interviewer collected data on adolescent sexual behavior are still widely utilized to inform policy and programming, including in the standard Demographic and Health surveys (DHS) which collect information from women starting at age 15. Randomization of the intervention strengthens the evidence that the effects observed between intervention and control arms are real and not due to selection bias. Further, the intervention was never framed to households as having the objective of HIV prevention and earlier evaluation rounds did not ask about HIV or sexual behavior, thus it is unlikely that intervention households would feel pressure to report socially desirable answers more than control households. Incident biologic endpoints clearly would strengthen the results of this study, however the costs of such measures precluded their inclusion in this study.

We have compared our sexual debut response rates with the 2008-09 Kenya DHS. Because our sample is somewhat unique in that it is based on the eligibility criteria for the CT-OVC, we compare our control sample with a DHS sample of people age 15–25 from the poorest two wealth quintiles living in the same geographical areas as our sample. We pool the two samples and use multivariate probit, controlling for age, sex and marital status of the respondent, to see if there is a difference in sexual debut rates between the two samples. We find that the sexual debut proportion is 5 percentage points lower in the DHS sample compared to our sample, which we believe is in the expected range given that our study protocols and instruments are similar to DHS but our study sample represents a potentially high risk group due to their unique demographic structure.

Another limitation of the study is the lack of pre-intervention sexual behavior data, thus we were unable to explore changes in behavior over time. However given that the intervention was randomized, differences observed between arms at wave three can be more strongly attributed to the intervention rather than other factors. In addition, the original evaluation design was not powered to study effects on sexual behavior, leading to small sample sizes for several of the secondary outcomes presented here. Finally, while early sexual debut is linked to increased risk of HIV infection in SSA, it is difficult to quantify the impact of the CT-OVC program on actual HIV incidence because not enough is known about how delayed sexual debut affects the risk of HIV infection (e.g., whether it is simply a marker for future HIV risk behaviors) [14] [16].

## Conclusion

We found that a major government social welfare program that is intended to alleviate poverty, the Kenya CT-OVC, reduces the likelihood of sexual debut by 23 percent among young people age 15–25. Importantly, this program did not focus on HIV or include any messaging about HIV prevention, and is similar to large scale social protection programs currently operating across Africa, thus adding to the evidence base that addressing upstream structural drivers of risk such as poverty can have important effects on reducing HIV risk. More work is needed to understand the mechanisms through which these programs reduce risk, and to determine if distinct mechanisms have differential relevance across outcomes. Rigorous evaluation of other large scale cash transfer programs will help elucidate the role of poverty alleviation programs in improving the health of populations, including in the arena of HIV prevention.

## Supporting Information

Table S1 **Determinants of Household Appearing in 2009 Survey Wave.**
(DOCX)
