# Supplementary material for: The Government of Kenya's Cash Transfer Program Reduces the Risk of Sexual Debut among Young People Age 15-25
Source: PLoS One. 2014 Jan 15;9(1):e85473. doi: 10.1371/journal.pone.0085473 (PMC3893206; doi:10.1371/journal.pone.0085473)
Supplement: Table S1 — Determinants of Household Appearing in 2009 Survey Wave. (DOCX) [file pone.0085473.s001.docx]

Table S1: Determinants of Household Appearing in 2009 Survey Wave

|  |  | Fully interacted model | |
| --- | --- | --- | --- |
|  |  | Main effect | Interactions with  treatment dummy |
|  | (1) | (2) | (3) |
| Treatment status | -0.0803^***^ | -0.252 |  |
|  | (0.0237) | (0.284) |  |
| Household size | -0.158^**^ | -0.329^**^ | 0.245 |
|  | (0.0573) | (0.116) | (0.134) |
| Residents 0-5 years | 0.0104 | 0.0387 | -0.0435 |
|  | (0.0128) | (0.0230) | (0.0282) |
| Residents 6-11 years | 0.0169 | 0.0545^*^ | -0.0575^*^ |
|  | (0.0122) | (0.0215) | (0.0265) |
| Residents 12-17 years | 0.0249^*^ | 0.0430^*^ | -0.0273 |
|  | (0.0122) | (0.0202) | (0.0255) |
| Residents 18-45 years | 0.0145 | 0.0340 | -0.0255 |
|  | (0.0111) | (0.0187) | (0.0235) |
| Residents 46-64 years | 0.0221 | 0.0436 | -0.0237 |
|  | (0.0218) | (0.0368) | (0.0460) |
| Residents 65+ years | 0.0278 | 0.0412 | -0.0155 |
|  | (0.0225) | (0.0391) | (0.0471) |
| Female head | -0.0282 | -0.0698^*^ | 0.0712 |
|  | (0.0207) | (0.0338) | (0.0427) |
| Age of head in years | -0.00447 | -0.00394 | -0.00164 |
|  | (0.00268) | (0.00390) | (0.00527) |
| Age of head in years^2^ (/100) | 0.00340 | 0.00306 | 0.00119 |
|  | (0.00194) | (0.00293) | (0.00390) |
| Head years of schooling | 0.00120 | -0.00108 | 0.00430 |
|  | (0.00254) | (0.00400) | (0.00506) |
| Per adult equiv. monthly exp. (Ks) (/100) | -0.000167 | 0.000150 | -0.000514 |
|  | (0.000254) | (0.00163) | (0.00174) |
| Walls of mud/dung/grass/sticks | 0.0293 | 0.0663 | -0.0397 |
|  | (0.0310) | (0.0539) | (0.0750) |
| Roof of mud/dung/grass/sticks | -0.0162 | 0.00852 | -0.0404 |
|  | (0.0292) | (0.0511) | (0.0552) |
| Floor of mud/dung | 0.00744 | -0.0270 | 0.0603 |
|  | (0.0238) | (0.0410) | (0.0491) |
| Cookfuel is firewood/residue/ | -0.00392 | 0.0538 | -0.113 |
| animal waste | (0.0305) | (0.0477) | (0.0714) |
| Has electric lighting | 0.0702 | 0.161 | -0.102 |
|  | (0.0658) | (0.0913) | (0.0546) |
| No toilet | 0.0408 | 0.0352 | -0.000162 |
|  | (0.0225) | (0.0315) | (0.0438) |
| Unprotected water source | 0.0555^**^ | 0.0110 | 0.0774 |
|  | (0.0198) | (0.0344) | (0.0452) |
| Crowding index | 0.0108 | 0.0234^*^ | -0.0187 |
|  | (0.00640) | (0.00944) | (0.0116) |
| Garissa | 0.0153 | -0.0353 | 0.132 |
|  | (0.0465) | (0.0608) | (0.119) |
| Homabay | 0.0349 | 0.00865 | 0.0489 |
|  | (0.0355) | (0.0446) | (0.0726) |
| Kisumu | 0.0859^**^ | 0.0718 | 0.0184 |
|  | (0.0313) | (0.0428) | (0.0559) |
| Kwale | 0.0338 | -0.0879 | 0.317^*^ |
|  | (0.0559) | (0.0530) | (0.157) |
| Migori | 0.0522 | -0.0142 | 0.108 |
|  | (0.0347) | (0.0440) | (0.0769) |
| Nairobi | 0.186^**^ | 0.262^*^ | -0.0525 |
|  | (0.0620) | (0.123) | (0.0829) |
| *N* | 2294 | 2294 | |

Dependent variable equals one if household appears in both 2007 and 2009 survey waves. Coefficients are marginal probabilities derived from probit estimates; standard errors in parentheses. ^*^ *p* < 0.05, ^**^ *p* < 0.01, ^***^ *p* < 0.001.
